# Immunotherapy Targeting Myeloid-Derived Suppressor Cells (MDSCs) in Tumor Microenvironment

**DOI:** 10.3389/fimmu.2020.585214

**Published:** 2021-02-04

**Authors:** Xidan Gao, Hongshu Sui, Shang Zhao, Xingmei Gao, Yanping Su, Peng Qu

**Affiliations:** ^1^ Department of Histology and Embryology, Shandong First Medical University & Shandong Academy of Medical Sciences, Taian, China; ^2^ Department of Pathophysiology, Shandong First Medical University & Shandong Academy of Medical Sciences, Taian, China; ^3^ Department of Neurology, People’s Hospital of Binzhou, Binzhou, China; ^4^ Center for Cancer Research, National Cancer Institute, Frederick, MD, United States

**Keywords:** cancer, tumor immunotherapy, tumor microenvironment, myeloid-derived suppressor cells, inhibitory factors

## Abstract

Myeloid-derived suppressor cells (MDSCs) are a heterogeneous population of immature myeloid cells that accumulate in tumor-bearing hosts to reduce T cells activity and promote tumor immune escape in the tumor microenvironment (TME). The immune system in the TME can be stimulated to elicit an anti-tumor immune response through immunotherapy. The main theory of immunotherapy resides on the plasticity of the immune system and its capacity to be re-educated into a potent anti-tumor response. Thus, MDSCs within the TME became one of the major targets to improve the efficacy of tumor immunotherapy, and therapeutic strategies for tumor MDSCs were developed in the last few years. In the article, we analyzed the function of tumor MDSCs and the regulatory mechanisms of agents targeting MDSCs in tumor immunotherapy, and reviewed their therapeutic effects in MDSCs within the TME. Those data focused on discussing how to promote the differentiation and maturation of MDSCs, reduce the accumulation and expansion of MDSCs, and inhibit the function, migration and recruitment of MDSCs, further preventing the growth, invasion and metastasis of tumor. Those investigations may provide new directions for cancer therapy.

## Introduction

Myeloid-derived suppressor cells (MDSCs), a population of immature myeloid cells with immunosuppressive roles in tumor-bearing models or patients with tumors, have been recognized as the major suppressor of the anti-tumor response (1, 2). The underlying mechanism and function of MDSCs in the TME have been studied by our team and other scientists (3, 4). Clinical data revealed that the high-level circulating MDSCs in patients with cancers correlated with clinical stage, metastatic burden, and the resistance to both chemotherapy and immunotherapy. MDSCs were also recognized as one of the major obstacles in the treatment of cancer, especially for tumor immunotherapy. Recently, epigenetic regulation of the biologic behavior of MDSCs had emerged as a promising tool in cancer therapy (5). Multiple strategies used to target these cells were investigated to determine if the immunosuppressive effects of MDSCs can be decreased or eliminated in order to improve the efficacy of anti-cancer immunotherapy. Even though no therapeutic drugs specifically targeting MDSCs had been approved to be used in clinical treatment, the effects of some agents had been evaluated in tumor mouse models and patients with cancer (6–9). Their roles were involved in promoting the differentiation and maturation of MDSCs, reducing the accumulation and expansion of MDSCs, preventing the migration and recruitment of MDSCs into tumor sites or/and metastasis areas, and impeding the suppressive function and activity of MDSCs (9). In this review, we summarized the data about the roles and functional mechanisms of those agents targeting MDSCs, further providing novel therapeutic strategies for clinical cancer treatment.

### Origin and Phenotype of MDSCs

Myeloid precursor cells, derived from hematopoietic stem cells (HSCs) in the bone marrow, developed into immature myeloid cells (IMCs) under physiological conditions. IMCs were differentiated into mature macrophages, dendritic cells (DCs), and granulocytes further. The different progenitor cells that formed the population, demonstrated a broad range of morphology and functional capacity. In contrast, in pathological conditions, such as inflammation, tumors, infections, or autoimmune diseases, there was the dramatic expansion of IMCs with the same phenotype and immune-suppressive activity, resulting in the further differentiation of IMCs into a large number of MDSCs in various tissues (10). Those MDSCs with potent immune-suppressive activity acted as negative regulators of those immune responses (11). Our data illustrated that during chronic process when inflammation transition into cancer, MDSCs downregulated anti-tumor immune responses by modulating cytokine production of macrophages and up-regulated the expression of immune-suppressive factors, such as arginase 1 (Arg-1) and inducible nitric oxide synthase (iNOS) (12, 13). Additionally, tumor MDSCs also blocked T cell anti-tumor response through the increase in the production of reactive oxygen species (ROS)/nitrogen (RNS) (14).

MDSCs lacked clear surface markers unlike monocytes, macrophages and DCs. According to surface markers, MDSCs were divided into two subsets, granulocytic/polymorphonuclear MDSCs (G-MDSCs/PMN-MDSCs) which their phenotype and morphology were similar to neutrophils, and the phenotype and morphology of monocytic MDSCs (M-MDSCs) were similar to monocyte. In tumor bearing-mice, MDSCs were characterized by co-expression of CD11b and Gr-1, which were further divided into two subtypes: CD11b^+^Ly6G^+^Ly-6C^high^ monocytic MDSCs (M-MDSCs) and CD11b^+^Ly-6G^+^Ly-6C^low^ polymorphonuclear MDSCs (PMN-MDSCs), which were usually present in bone marrow, peripheral blood, spleen, liver, lung or various organs (14). In Human, M-MDSCs were defined as CD11b^+^CD14^+^HLA-DR^−/lo^CD15^−^ while PMN-MDSCs were defined as CD11b^+^CD14^−^CD15^+^ or CD11b^+^CD14^−^CD66b^+^ (4). Those phenotypes and function of MDSCs had been shown in our recent articles (3, 15). Both PMN-MDSCs and M-MDSCs had different immunosuppressive mechanisms (16).

### Regulatory Mechanism of MDSCs Within the TME

**Figure 1:**
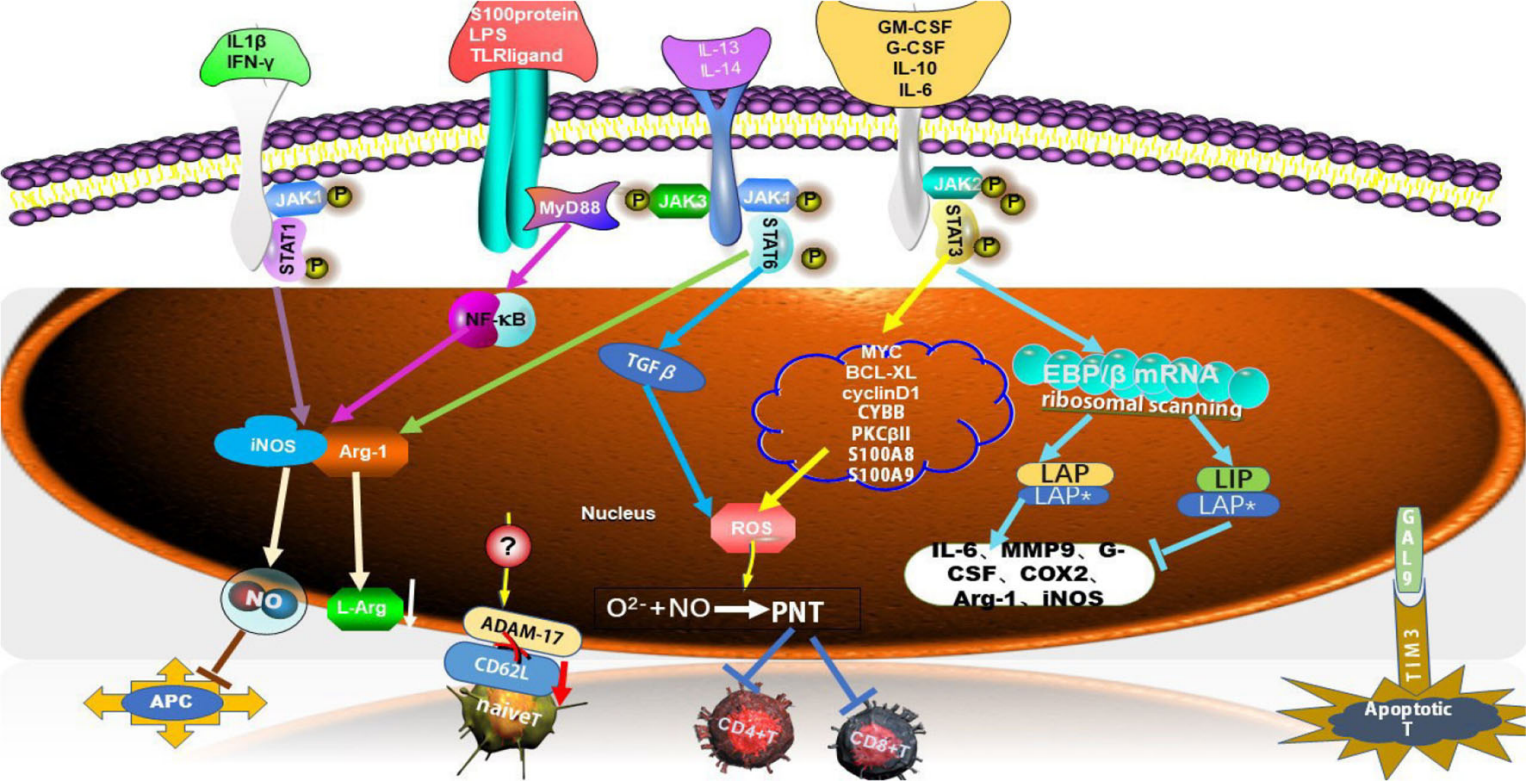
Multiple MDSC-mediated immunosuppressive mechanisms. MDSCs suppressed T cell function through multiple mechanisms. Several factors were involved in triggering signaling pathway, such as STAT1, STAT3, STAT6 and MyD88, which led to high expression level of immunosuppressive factors, such as Arg-1, iNOS, ROS, NO, which suppress T cells response. C/EBPβ was one of the family of CCAAT/enhancer-binding proteins (C/EBPs) which belonged to transcription factors (TFs), which had three isoforms, LAP*, LAP and LIP. S100A8 and S100A9 along with gp91phox (also known as CYBB) were part of the NADPH oxidase (NOX) complex that was responsible for the increased production of reactive oxygen species (ROS) in MDSCs. ADAM17, disintegrin and metalloproteinase domain 17; JAK, Janus kinase; STAT, signal transducer and activator of transcription; PNT, peroxynitrate; COX2, cyclooxygenase-2; APC, antigen-presenting cell; MMP9, matrix metalloproteinase 9; MyD88, myeloid differential protein-88; G-CSF, granulocyte colony-stimulating factor; GM-CSF, granulocyte-macrophage colony stimulating; BCL-XL, B-cell lymphoma XL; GAL9, galectin 9; TIM3, T cell immunoglobulin and mucin domain-containing protein 3.

One of the main features of MDSCs was to exhibit immunosuppression activity which was involved in multiple mechanisms or/and factors including signal transducer and an activator of transcription (STATs) (such as STAT1,STAT3, and STAT6) (17), some cytokines (IFN-γ, IL-10, IL-6, GM-CSF), and special molecules (PGE2, S100 protein, and LPS, etc.) (18). MDSCs also hindered the anti-tumor roles of many immune cells in the immune system, such as Natural Killer (NK) cells, B cells and T cells. And the inhibition of T cell function was most important for evaluating the activity of MDSCs. In TME, MDSCs depleted the essential nutrients of T cells through STAT/MyD88 signaling pathway to up-regulate metabolic enzymes e.g. Arg-1, iNOS, which reduced the expression of l-arginin, a substance necessary for T cell activation and proliferation (17) (**Figure 1**). Thus, l-arginin metabolism played a key role in the immunosuppressive activity of MDSCs by altering the mRNA transcription (19). l-arginin was metabolized by inducible iNOS, generating citrulline and NO to suppress T cell activation and decrease MHC II molecular expression on antigen-presenting cells (APCs), and further inducing T cell apoptosis (18). The productions of ROS/RNS which MDSCs produced, were also immunosuppressive necessary factors. The action of NO and O^2−^ produced peroxynitrite (PNT), which directly impaired T cell activation by nitrating T cell receptors and reducing the reactivity of MHC antigen complexes. PNT reduced the integration of MHC I molecules with antigenic peptides on tumor cells and nitrified T cell-specific chemokines to prevent T cell migration (17, 19–21) (**Figure 1**).

Apart from the suppression roles on T cells, MDSCs also induced T cells apoptosis and blocked T cell migration which was essential for T cell responses. Galectin 9 expressed on MDSCs bond to T cell immunoglobulin and mucin domain-containing protein 3 (TIM3) on lymphocytes and induced T cell apoptosis (19) (**Figure 1**). MDSCs directly down-regulated the expression of CD62L on naïve T cells through the expression of TNF-α-converting enzyme (TACE/ADAM17) on MDSCs to hamper the homing of naïve T cells to lymph nodes, leading to the reduced number of those cells (22).

MDSCs and regulatory T (Treg) cells are major components of the TME. Both cell types expanded in tumor models or patients with cancer and promoted T cell dysfunction that in turn facilitated tumor progression (23). Recent studies revealed that MDSCs promoted the development and induction of Treg cells to enhance their suppressive roles on T cells (24). In Ret transgenic mouse models (melanoma model), tumor M-MDSCs also drove the recruitment of CCR5+ Treg cells through producing chemokines such as CCL3, CCL4 and CCL5, further accelerating tumor metastasis (25) (**Figure 1**).

### Immunotherapy Through Targeting Tumor MDSCs

The suppressive roles of MDSCs on both T cells response and the functions of multiple types of cells are critical for the anti-tumor immune response. MDSCs accumulated within the TME were recognized as a major obstacle for tumor immunotherapy (3, 15). Therefore, there are increasing evidences to show that it may be novel therapy strategies for tumor to identify inhibitory factors and find therapeutic ways on tumor MDSC. In the review, we summarized recent data about therapeutic agents and methods on tumor MDSCs, based on the functional characterization of MDSCs.

#### Promote the Differentiation and Maturation of MDSCs

Within the TME, more IMCs were differentiated into a large number of MDSCs, whereas further differentiation of MDSCs into mature macrophage or DCs was restrained. The agents that promoted differentiation and maturation of MDSCs had been investigated as potential therapeutic strategies to reduce or eliminate MDSCs. Those agents, including all-trans retinoic acid (ATRA), IL-12, RUNX1, and CpG oligonucleotides drove MDSCs differentiation into mature myeloid cells (26–29).

Both Docetaxel and Paclitaxel treated breast cancer effectively, as the two clinical representatives of the new class of taxane drugs. Recent reports demonstrated that Docetaxel promoted MDSCs differentiation into M1-like macrophages with anti-tumor activity by reducing the phosphorylation levels of STAT3 in 4T1 breast tumor-bearing mice (30). And Paclitaxel promoted tumor MDSCs differentiation into mature DCs in a TLR4-independent manner (31) (**Table 1**).

**Table 1:** The immunoregulatory agents target MDSCs within the TME.

Tumor types	Agents	Mechanisms/Functions	Advantage	Disadvantage	Reference
4T1 breast cancer (mouse model)	Docetaxel	To polarize MDSC differentiation into M1-like macrophages through the reduced phosphorylation of STAT3.	Direct effect of chemotherapeutic agents on tumor and tumor MDSCs	N/A	(30)
In vitro MDSC culture model	Paclitaxel	To promote MDSC differentiation into DCs,	Ultra-low non-cytotoxic doses of paclitaxel induces MDSC differentiation	N/A	(31)
Breast/colonic cancer; renal carcinomas (mouse models)	Sunitinib	To reduce the number of MDSCs through inhibition of STAT3 signal.	To target both cancer cells and MDSCs	N/A	(32)
Renal cell carcinoma (patients)	Sunitinib	Blockade of VEGF and c-KIT signal	To diminish the number of both MDSCs and Treg cells	No correlation between a change in tumor burden and a change in MDSCs/Treg	(33)
Melanoma (mouse model)	DATS	To abrogate number and immunosuppressive activity of MDSCs.	To improve T cell anti-tumor response.	N/A	(34)
CLL (mouse model)	Vitamin D	To downregulate MDSC function as negative regulator of miR155.	To easily enhance anti-tumor activity	N/A	(35)
Gastric/colonic cancer (mouse models)	Curcumin	To inhibit the functions of MDSCs by the inactivation of STAT3 and NF-kB signaling	To interfere with the interaction between cancer cells and MDSCs	N/A	(7)
Head/neck squamous cell carcinoma (patients)	Tadlafil	Inhibitors of PDE5. To inhibit the activity of iNOS and Arg-1to reduce both MDSCs and Treg concentrations	To promote the activation of CD8+ T cells at the tumor site	Grade 1–3 adverse events (such as back pain/myalgia)	(36)
Pancreas/lung cancer (mouse models)	Entinostat	To neutralize MDSCs through reduced expression of Arg-1, iNOS, and COX2.	To enhances the antitumor effect of PD-1	N/A	(37, 38)
NSCLC (patients)	JNJ-61610588	Anti-VISTA monoclonal antibody	To inhibit the function of both Treg cells and tumor MDSCs	To induce autoimmunity	(39)
Metastatic melanoma/breast cancer/NSCLC (patients)	Pembrolizumab	PD-1blocker	To inhibit both tumor and tumor MDSCs	To induce pneumonitis	(40)
Melanoma (patients)	Nivolumab Lambrolizumab	Monoclonal antibodies targeting PD-1	To result in a high rate of sustained tumor regression	Grade 1-2 toxic effects (including diarrhea, nausea)	(41, 42)
Metastatic urothelial carcinoma (patients)	Atezolizumab	PD-L1 monoclonal antibody	To inhibit PD-L1 positive cells (tumor cells and MDSCs)	Grade 3–4 adverse events (including pneumonitis/fatigue)	(43)
Metastatic melanoma (patients)	Ipilimumab	Human monoclonal antibody against CTLA-4	To boost the body’s immune response against cancer cells	To induce diarrhea	(44)

CLL, chronic lymphocytic leukemia; APCs, antigen-presenting cells; DATS, diallyl trisulfide; DCs, dendritic cells; PDE5, phosphodiesterase-5; N/A, not available; TME, tumor microenvironment; NSCLC, non-small cell lung cancer.

**Figure 2:**
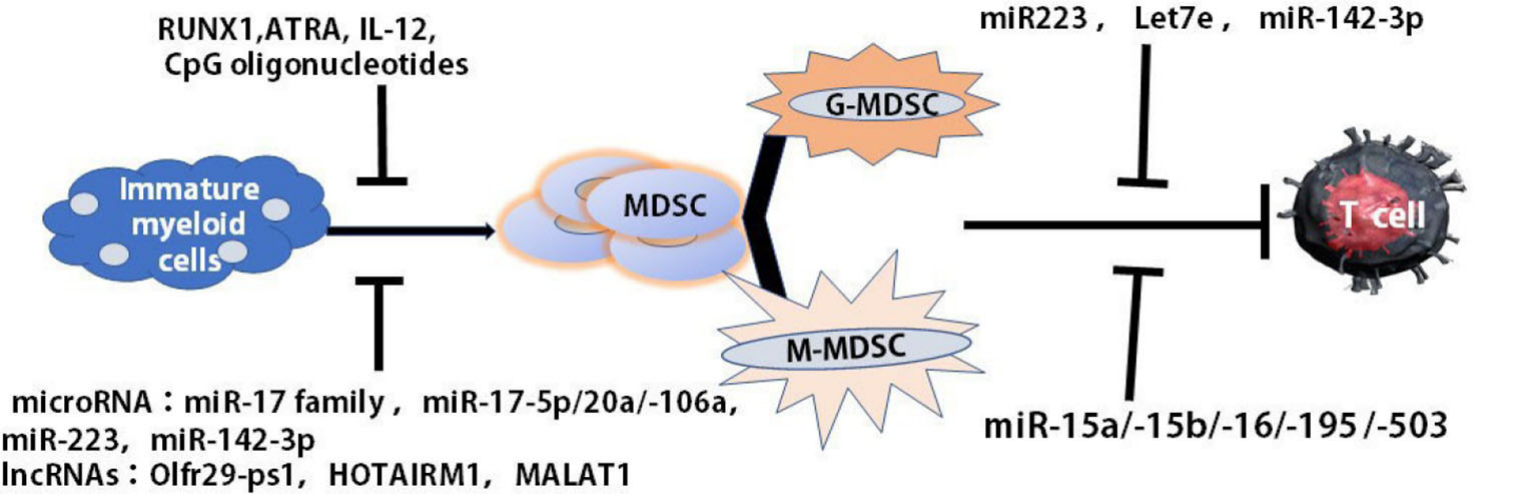
Multiple agents regulated the maturation and differentiation of MDSCs. The differentiation and maturation of MDSCs were regulated by multiple agents, such as RUNX1, ATRA, IL-12, CCL5, and CpG oligonucleotides as well as miRNA, lncRNA. ATRA, all-trans retinoic acid (ATRA).

The differentiation and function of MDSCs may be regulated through microRNA (miRNAs), Long non-coding RNAs (LncRNAs) and epigenetic modifying factors. Our recent data demonstrated that the differentiation and maturation of tumor MDSCs were mediated miRNAs which participated in regulating cell proliferation, differentiation, and maturation (45). The overexpression of miR-17 family members, such as miR-17-5p, miR-20a, and miR-106a in human progenitor cells repressed AML1 by binding to its promoter to down-regulate M-CSFR which induced MDSCs differentiation. MiR-223 remarkably prevented the differentiation of IMCs into MDSCs in the presence of tumor-associated factors by targeting myocyte enhancer factor 2C (MEF2C). MiR-142-3p could limit the generation of MDSCs during tumor-induced myelopoiesis by modulating STAT3 and CCAAT/enhancer-binding protein β (C/EBPβ) signal pathways (45, 46) (**Figure 2**).

LncRNAs, more than 200 introns, were intergenic, intronic and natural antisense transcripts, or transcribed from divergent enhancers and promoters (47). They modified chromatin, adjusted the networks of genetic and signal pathways in the pathogenesis of cancer and played a critical role in the regulation of the function and development of myeloid cells (48, 49). Some of the pseudogene transcripts could function as LncRNAs to regulate related gene expression by different mechanisms (50) (**Figure 2**). Olfr29-ps1, a LncRNA pseudogene, expressed highly in MDSCs, downregulated miR-214-3p to promote the differentiation of MO-MDSCs through IL6-mediated N6-methyladenosine (m6A) modification manner (51). Metastasis associated lung adenocarcinoma transcript 1 (MALAT1), as a nuclear intergenic of LncRNA, whose expression was involved in the differentiation of MDSC-like cells. The reduced expression of MALAT1 in the patients with lung cancer led to the increased proportions of MDSCs, indicating that MALAT1 could prevent the differentiation of tumor MDSCs (52) (**Figure 2**). HOXA transcript antisense RNA myeloid-specific 1 (HOTAIRM1), an intergenic lncRNA localized between homeobox (HOX)A1 and HOXA2 genes, was expressed preferentially in the myeloid lineage and was a key regulator that targets HOXA1 during myeloid cell development. HOTAIRM1 promoted the maturation of MDSCs *via* inducing HOXA1 expression in MDSCs to retard lung cancer growth (53). Therefore, multiple agents were found to regulate the maturation and differentiation of MDSCs (**Figure 2**).

#### Reduce the Accumulation and Expansion of MDSCs

MDSCs were not present in the circulatory system under normal physiological conditions. However, these cells accumulated in the individuals with cancer. Many agents participated in inducing the decrease of tumor MDSCs. The liver X receptor (LXR) beta-agonist could diminish the populations of both granulocytic and monocytic MDSCs in multiple mouse cancer models by inducing MDSCs apoptosis, which was mediated through the LXR target gene, ApoE, which bond to the LRP8 receptor on the surface of MDSCs (54).

AMP-activated protein kinase (AMPK), an important protein kinase, regulated energy metabolism and innated adaptive immunity by targeting the major signaling pathways. AMPK activation could diminish the expansion and activation of MDSCs through inhibiting multiple signaling pathways, such as STATs and NF-κB pathways (55, 56), and alleviated the nuclear translocation of STAT1 through the increased expression of mitogen-activated protein kinase phosphatase-1 (MKP-1) (57, 58). AMPK also decreased the expansion of MDSCs through attenuating NF-κB activation as well as oxidative and endoplasmic reticulum (ER) stresses, since NF-κB signaling played an important role in the expansion of MDSCs in tumor initiation and progression (59). As a receptor tyrosine kinase inhibitor, Sunitinib was approved by the FDA for the treatment of metastatic renal cell carcinoma (RCC). Recently, some scientists found that Sunitinib was also an immune-modulator, potently reversing tumor MDSC accumulation through STAT3 or C-Kit signal (32, 33) (**Table 1**).

H2S was an endogenous signaling molecule with a plenty of cellular and molecular targets, which were produced in various mammalian cells and tissues by three principal enzymes: cystathionine-β synthase (CBS), cystathionine-γ lyase (CSE), and 3-mercaptopyruvate sulfurtransferase (3-MST). In melanoma bearing mice, administration of Diallyl Trisulfide (DATS) — H2S donor and the activator of CSE, reduced the number of MDSCs in spleen and blood through the reduction of iNOS expression and NO production. The functional mechanism was involved in the endogenous production of H2S which up-regulated the expression levels of both CSE and CBS to decrease the number of MDSCs in the TME. In addition, DATS also induced MDSCs differentiating into mature APCs (34) (**Table 1**).

The accumulation of tumor MDSCs was also mediated by miRNA, such as miR-223 and miR-155 (60). miR-155, which was processed from the B-cell integration cluster (BIC), triggered the differentiation of CD4+ T cells into the Th1, Th2, and Th17 pathways and was required for CD8+ T cell responses to acute viral and bacterial challenges (61–63). In the TME, miR-155 promoted the accumulation of functional MDSCs through the SOCS-1 repression, facilitating tumor growth (64). In chronic lymphocytic leukemia (CLL), MDSC accumulation upregulated by exosome miR-155 was disrupted by vitamin D, one negative regulator of miR155, which enhanced anti-tumor activity (35) (**Table 1**).

#### Abrogate MDSC Immunosuppression

**Figure 3:**
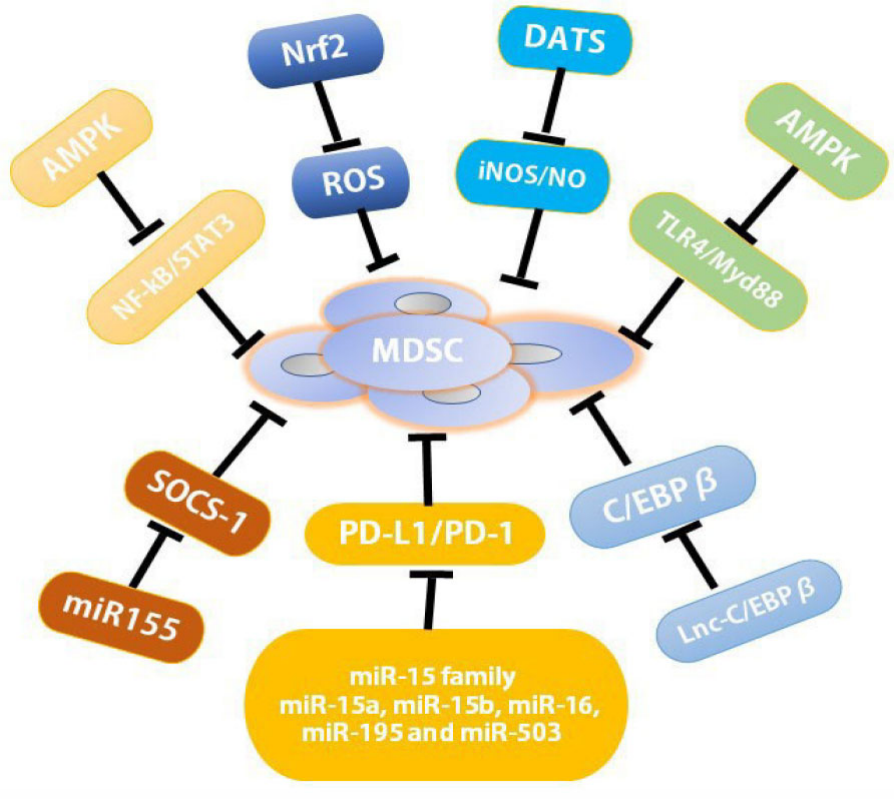
Resistance mechanisms to MDSC immunosuppression. AMPK activation inhibited the TLR4/Myd88 pathway as well as STATs and NF-κB pathways to attenuate the immunosuppressive function of tumor MDSCs; miR-15 family including miR-15a, miR-15b, miR-16, miR-195, and miR-503, down-regulated the suppressive function of MDSCs and/or Tregs within the TME through blocking PD-L1/PD-1 signaling pathway; miR-155 regulated tumor MDSC *via* the repression of SOCS-1 in MDSCs; Nrf2 activation reduced intracellular ROS production to abrogate MDSC immunosuppression; DATS, as H2S donors and an activator of CSE reduced the number of MDSCs in spleen and blood through the inhibition of iNOS expression and NO production; Lnc-C/EBPβ mastered suppressive functions and differentiation of MDSCs by binding to C/EBPβ (specifically to the LIP isoform). AMPK, AMP-activated protein kinase; C/EBPs, CCAAT/enhancer-binding proteins; miRNA, MicroRNA; LncRNAs, long non-coding RNAs; MALAT1, metastasis associated lung adenocarcinoma transcript 1; DATS, diallyl trisulfide.

The inhibition of MDSC immunosuppression function is the major therapeutic target to re-establish T cell activity in cancer immunotherapy. One of the well-known pathways was the JAK-STAT3 signaling pathway which played the crucial role in the regulation of immune responses and induction of the immune escape of tumors through regulating the expression of cytokines and growth factors, further regulating the function of MDSCs (65) (**Figure 3**). Both STAT3 and NF-κB signaling were essential for the attenuation of Curcumin on the functions of MDSCs (7). AMPK activation phosphorylated the protein to block the activity of Janus kinase 1 (JAK1) to suppress STAT3-dependent gene expression directly, further dampening the suppressive function of MDSCs (66).

Besides reducing the accumulation and expansion of MDSCs, AMPK activation also attenuated the immunosuppressive function of tumor MDSCs through TLR4/Myd88 pathway (67–69). Metformin, one anti-diabetes drug broadly used for the treatment of type 2 diabetes, might act as a new anti-cancer drug since it reduced the immunosuppressive activity of MDSCs by triggering the activation of AMPK (70, 71) (**Figure 3**). Metformin down-regulated the function of G-MDSCs through AMPK/STAT3 pathways, delaying tumor progression in CT-26 cell colon cancer mouse model (71). In patients with ovarian cancer (OC), Metformin suppressed hypoxia-inducible factor-α (HIF-1α) which blocked CD39/CD73 expression in MDSC to impair MDSC function (72) (**Figure 3**).

MDSCs upregulated the expression of immune suppressive factors such as ROS, iNOS and Arg-1 to reduce T cells anti-tumor activity. Thus, those factors above became important therapeutic targets. Nuclear factor erythroid 2-related factor 2 (Nrf2) modulated the expression of antioxidant enzymes, including NADPH, NQO1, Hem oxygenase to confer cryoprotection against oxidative stress (73). Selective activation of Nrf2 reduced intracellular ROS production to abrogate MDSC immunosuppression, further reducing tumor metastasis. The promising anticancer drugs had been tested in Phase 1 clinical trials of patients with tumors (73–75). Moreover, Tadalafil was found to reduce activity of iNOS and Arg-1 on both MDSCs and Tregs to disrupt their roles in patients with head/neck squamous cell carcinoma (HNSCC), even though there were low grade of adverse events (such as back pain/myalgia) (36). Entinostat, as class I histone deacetylase inhibitor (HDAC), neutralized MDSCs through reducing the expression of both Arg-1 and iNOS in mouse models of pancreas/lung cancer (37, 38) (**Table 1**).

Checkpoints were divided into stimulatory and inhibitory forms, which precisely regulated T cell activation. Its balance-maintained self-tolerance and prevented autoimmunity. The upregulation of inhibitory checkpoints led to T cell exhaustion by inhibiting TCR and interactions between co-stimulatory molecules and ligands present on APCs (39). The inhibitory checkpoints included cytotoxic T lymphocyte–associated antigen 4 (CTLA-4), programmed death 1 (PD-1), and V-domain Ig suppressor of T cell activation (VISTA) etc. CTLA-4 were expressed on T cells and bond to CD80/CD86 to mediate the inhibition of Tregs.) (76). PD-1 regulated T cell activation through binding to its ligands, programmed death ligands 1 (PD-L1, B7-H1, or CD274) or 2 (PDL-2, B7-DC, or CD273). The immunotherapy of PD-L1/PD-1 on tumor has been broadly applied. There were increasing evidence that immunosuppression function of MDSCs was mediated by PD-L1 (77). PD-L1 was constitutively expressed and inducible in tumor cells and tumor MDSCs, and bond to PD-1 to suppress T cell activation as an inhibitory ligand. In mouse bladder cancers, PD-L1 expression on tumor-associated MDSCs was associated with the expression of cyclooxygenase-2 (COX2), microsomal prostaglandin E (mPGES1), and prostaglandin E2 (PGE2), as well as their capacity to induce apoptosis of CD8+ T cells (78). The expression of PD-L1 on MDSCs was upregulated in response to multiple microenvironment signals, including hypoxia *via* HIF1α, and IFN-γ *via* the STAT1/IRF1 axis (79). In addition, the interaction between PD-L1/PD-1 and miRNAs was required for the function of tumor MDSCs (77). Our recent report revealed that five members of the miR-15 family, including miR-15a, miR-15b, miR-16, miR-195, and miR-503, down-regulated the suppressive function of MDSCs and/or Tregs in the TME through blocking PD-L1/PD-1 signaling pathway (45). The expression of PD-L1 on tumor MDSCs was modulated by the miR-93/106b miRNA cluster of miR-17 family through the STAT3 pathway. Those PD-L1 expression levels on MDSCs could be reduced significantly after the treatment of miR-93 mimics (9, 45). Thus, these PD-1/miRNA/STAT3 pathways provided a new idea of treatment idea for hindering MDSC-associated tumor metastasis (80) (**Table 1**).

VISTA, another negative checkpoint regulator in the B7 family, enhanced Treg maturation and inhibited T cell activation and hence contributed to the TME (81–83). Its blockade led to the decreases of both Treg cells and MDSCs in the TME, activated DCs, inducing tumor regression in AML mouse models (84–86) and human oral squamous cell carcinoma (OSCC) (87, 88). Recently, the drugs targeting CTLA-4, PD-1, PD-L1, and VISTA were approved to inhibit tumor growth and metastasis, even though these drugs had adverse events for patients with cancer during the treatment (40–44) (**Table 1**).

LncRNAs also participated in regulating the function of MDSCs through mediating gene transcription. Multiple LncRNAs have been described in myeloid derive cells. Recently, Lnc-C/EBPβ was identified to master suppressive functions of MDSCs by binding to C/EBPβ (specifically to the LIP isoform). C/EBPβ was one of the families of C/EBPs—a group of transcription factors (TFs), which had three isoforms, LAP*, LAP, and LIP, with the former one mainly functioning as transcriptional activator of the expression of immunosuppressive genes such as Arg-1, NOS2, NOX2, or COX2 (89). After exogenous Lnc-C/EBPβ treatment, these immunosuppressive factors above were decreased in quantity, indicating that Lnc-c/EBPβ may play a negative regulatory role in the immunosuppressive function of MDSCs (90) (**Figure 3**). LncRNA Pvt1 expression in G-MDSCs was upregulated under hypoxia which was a typical feature within the TME. LncRNA Pvt1 knockdown led to the decreased suppression of G-MDSCs, partially restoring T cell antitumor responses (91). In humans, the overexpression of LncRNA PVT1 was tightly associated with a variety of cancer types, including hepatocellular carcinoma, gastric cancer, esophageal cancer, and acute myeloid leukemia (92–97) (**Figure 3**).

#### Prevent the Migration and Recruitment of MDSCs

MDSCs exhibited their immunosuppressive activity mainly within the TME. Therefore, the intensive investigations were also conducted to block the migration of MDSC to the tumor sites. PGE2 was involved in tumor angiogenesis and progression *via* the recruitment of MDSCs (98–100). The inactivation of COX‐2/PGE2 signaling succeeds in reducing MDSC recruitment to retard tumor growth (101). CSF‐1R, a tyrosine kinase receptor, was also involved in the migration of MDSCs (8). Treatments targeting the receptor or its ligand CSF‐1R/CSF‐1 were found to prevent MDSC recruitment to the tumor site to improve T‐cell activity. In addition, anti-glycan antibodies designed to target the receptor for advanced glycation end products (RAGE) were revealed to prevent the recruitment of MDSCs to cancer areas through the S100A8/A9 feedback loop (8).

The chemokines and chemokine receptors played important roles in the migration of tumor MDSCs. The chemokine receptors on MDSCs as therapeutic targets have been used to prevent the recruitment of MDSCs to the tumor sites or metastatic areas. MDSCs were driven into the TME through the chemokines receptor CCR5 expressed on MDSCs with the help of the ligands CCL3, CCL4, and CCL5 (102). In melanoma mouse models and human patients, MDSCs that expressed CCR5 were found to have more potent immunosuppressive effect compared to those that did not express CCR5. Blattner et al. demonstrated that the blockade of CCR5 dampened the recruitment and immunosuppressive activity of MDSCs and improved survival rate (103). Elevated level of CCL2 and CCL5 were present in the TME to recruit MDSCs through chemokine receptor CXCR2 (104, 105). CXCR2+ MDSCs promoted tumor expansion and metastasis in breast cancer (106). By targeting CXCR2, MDSCs were diminished to block tumor metastasis, promoting T‐cell infiltration into the tumors and extending survival in pancreatic cancer (107). Metformin reduced CXCL1 secretion in esophageal squamous cell carcinoma (ESCC) cells through enhancing AMPK phosphorylation and inducing Dachshund homolog 1 (DACH1) expression. Knockdown of both AMPK and DACH1 blocked the effect of metformin on MDSC chemotaxis, indicating that AMPK-DACH1-CXCL1 axis played a significant role in Metformin-regulated migration of MDSCs (70). In summary, targeting chemokine receptors on MDSCs could be applied to prevent the migration and accumulation of MDSCs in the TME.

## Conclusion and Perspectives

Tumor MDSCs were recognized as a major obstacle for tumor immunotherapy, As those results, many scientists continue looking for the inhibitory products targeting tumor MDSCs and evaluating their effects to develop new therapeutics that improve the efficacy of cancer immunotherapy strategies. While many of the cancer immunotherapies focus on the manipulation of T cells, the therapy targeting MDSCs may provide another idea for anti-tumor treatment.

MDSCs are major functional cells in the TME through multiple mechanisms to facilitate the harnessing of anti-tumor response. The radiolabeled MDSCs imaging approach to visualize MDSCs migration and tumor homing *in vivo* is used to further analyze the effects of cancer therapies and immune-therapeutics on MDSC migration and their capacity to infiltrate tumors (108). In addition, MDSCs from patients with tumor are quantified as one marker to differentiate patients with active vs inactive cancer-including radiation necrosis, using liquid biopsies (109). Those results indicated that MDSCs may also one useful clinical biomarker for evaluating radiotherapy effect and identifying tumor metastasis.

## Author Contributions

XDG wrote, reviewed, and revised manuscript, figures, and tables. HS prepared the figures and tables. SH reviewed and revised the manuscript. YS revised the manuscript. PQ wrote, reviewed, and revised manuscript, figures, and tables. All authors contributed to the article and approved the submitted version.

## Funding

This work was supported by the National Natural Science Foundation of China (grant 81572868); Science Foundation of Shandong (grant ZR2018LC012).

## Conflict of Interest

The authors declare that the research was conducted in the absence of any commercial or financial relationships that could be construed as a potential conflict of interest.
